# Case Report: Meningoencephalomyelitis of unknown origin in foxes (*Vulpes vulpes* and *Vulpes lagopus*): insights into the diagnostic challenges in carnivore neurology

**DOI:** 10.3389/fvets.2025.1630662

**Published:** 2025-08-21

**Authors:** Natalie Steiner, Martin Dembowski, Florian Richard Moeselaken, Monica Mirolo, Martin Ludlow, Julie-Ann Dierig, Manon Mikić, Antja Watanangura, Reiner Ulrich, Christina Puff, Michael Pees, Jasmin Nicole Nessler

**Affiliations:** ^1^Department of Small Mammal, Reptile and Avian Diseases, University of Veterinary Medicine Foundation, Hannover, Germany; ^2^Faculty of Veterinary Medicine, Institute of Veterinary Pathology, Leipzig University, Leipzig, Germany; ^3^Department of Pathology, University of Veterinary Medicine Foundation, Hannover, Germany; ^4^Research Center for Emerging Infections and Zoonoses, University of Veterinary Medicine Foundation, Hannover, Germany; ^5^Department of Small Animal Medicine and Surgery, University of Veterinary Medicine Foundation, Hannover, Germany

**Keywords:** diagnostic challenges, non-infectious meningoencephalomyelitis, immune-mediated disease, wildlife health, case report

## Abstract

Emerging diseases in wildlife pose significant diagnostic challenges, with increasing evidence that not all cases of inflammatory disease can be directly attributed to infectious pathogens. This case series shows the results of clinical examination, magnetic resonance imaging, and necropsy results of two foxes, a silver fox (*Vulpes vulpes*) and a polar fox (*Vulpes lagopus*), with non-suppurative meningoencephalomyelitis. Extensive diagnostics, including pathogen screening and next-generation sequencing, failed to identify a definitive causative infectious agent. These cases suggest the possibility of an immunopathologic disease, highlighting the need for immunological assessments in wildlife disease investigations.

## 1 Introduction

Meningoencephalomyelitis is commonly observed in foxes (*Vulpes* spp.) (1, 2). Despite viruses, bacteria, and parasites being the most commonly reported causes of meningoencephalomyelitis (1, 3), several studies have reported wild carnivores with meningoencephalitis without identifiable pathogen (1–4). These findings raise concerns about the limitations of current diagnostic methods, the potential involvement of undetected pathogens, and the possibility of immune-mediated mechanisms contributing to CNS inflammation. A similar diagnostic challenge exists in domestic species: in dogs and cats, non-suppurative meningoencephalomyelitis of unknown etiology is often classified under the umbrella term meningoencephalomyelitis of unknown origin (MUO) (5). The pathogenesis of MUO remains a topic of considerable debate, with prevailing theories suggesting a combination of genetic predisposition and environmental triggers that provoke an exaggerated immune response, potentially influenced by an infectious agent (5, 6). The favorable response to immunosuppressive treatments further supports the hypothesis of an immune-mediated disease in domestic dogs (7).

The following case report presents the diagnostic and necropsy results of two foxes with meningoencephalomyelitis where despite extensive investigation no infectious agents could be identified.

## 2 Materials and methods

Over a period of 3 years, 12 foxes (two polar and 10 silver foxes) rescued from Polish fur farms and housed in a single facility developed neurological disorders and subsequently died. One fox died without exhibiting clinical signs. In two cases, a diagnostic workup was performed; for the remaining cases, no additional diagnostic information was available beyond what is presented here, as further testing was not conducted or accessible.

### 2.1 Clinical evaluation

Following clinical examination, further diagnostic procedures were conducted under general anesthesia. Anesthesia was induced intramuscularly using ketamine (10 mg/kg; Ketamin 100 mg/mL), butorphanol (0.3 mg/kg IM; Butorgesic 10 mg/mL, and medetomidine; 15 μg/kg IM; Cepetor 1 mg/mL; all CP-Pharma Handelsgesellschaft GmbH, Burgdorf, Germany), with maintenance using alfaxalone (1 mg/kg intravenous; Alfaxan® Multidose 10 mg/mL, Jurox Animal Health, Rutherfort, Australia) and isoflurane in an oxygen/air mixture (1:1, flow 50 mL/kg/min). A venous catheter was placed in the *vena cephalica*, and orotracheal intubation was performed.

### 2.2 Laboratory analysis

Blood was sampled for a complete blood count using the ADVIA 120 Hematology System (Siemens Healthcare GmbH, Erlangen, Germany), blood smear examination via manual microscopy (Diff-Quick Staining solution, Merck, Darmstadt, Germany), biochemistry via the cobas c 311 analyzer (Roche Germany Holding GmbH, Mannheim, Germany), and electrolyte levels using the RAPIDLab 1260 (Siemens Healthcare GmbH). Urine analysis was performed via human urine dipsticks (Combur 9 Roche Germany Holding GmbH). Urinary density was measured via refractometer.

Blood for blood culture was collected directly from the heart immediately after death.

### 2.3 Imaging

Radiographic examinations of the thorax and abdomen were conducted with a PHILIPS Bucky Diagnost TH AGFA DX-S system, and whole-body computed tomography (CT) imaging was carried out using the Philips IQon Spectral CT scanner (Philips Medical Systems, Best, The Netherlands). Magnetic resonance imaging (MRI) of the head and thoracolumbar vertebral column was performed with a 3.0 T Achieva MRI scanner (Philips Medical Systems, Best, The Netherlands), including T2-weighted (T2w), fluid-attenuated inversion recovery (FLAIR) and pre- and post-contrast T1-weighted (T1w) sequences. Cerebrospinal fluid (CSF) was collected suboccipital from the cisterna magna in lateral recumbency after clipping and aseptic preparation. CSF analysis included manual cell count via Fuchs-Rosenthal-chamber, manual cell differentiation, and measurement of glucose, protein, and albumin using commercially available photometric assay tests (cobas c311 analyzer, Hitachi, Roche, Mannheim, Germany), adapted for dogs.

Reference values for blood samples were based on previously published values for red foxes (8). Reference values for urine and CSF analysis were taken from domestic dogs.

### 2.4 Postmortem examination

Fox No. 1 died under anesthesia and fox No. 2 was euthanized using pentobarbital (1 mL/kg intravenous (IV); Narkodorm® 200 mg/mL, CP-Pharma Handelsgesellschaft GmbH, Burgdorf, Germany).

Tissue samples were collected from the brain, spinal cord, peripheral nerves, lung, liver, spleen, kidneys, heart, eyes, thyroid, and others, for histological, histochemical, and immunohistochemical analyses. The tissues were fixed in 10% neutral buffered formaline for at least 24h, embedded in paraffin, and sectioned to approximately 2 μm. Hematoxylin and eosin (HE) staining was performed on tissue sections, which were then examined under an Olympus BX53 microscope (Olympus Europa SE & Co. KG, Hamburg, Germany), and images were captured using an Olympus DP28 camera (Olympus Europa SE & Co. KG, Hamburg, Germany).

### 2.5 Pathogen screening

For fox No. 1, blood samples were tested for antibodies against Tick-Borne Encephalitis Virus (TBEV), Morbillivirus, *Encephalitozoon cuniculi*, *Borrelia* spp.*, Toxoplasma gondii*, and *Neospora caninum*. Polymerase chain reaction (PCR) for *Leptospira* spp. was performed on EDTA blood. CSF was tested for antibodies against TBEV, *Neospora caninum*, and *Canine adenovirus 1.* PCR assays were performed for *Anaplasma phagocytophilum, Neospora caninum*, and *Toxoplasma gondii* in CSF. Bacterial cultures were performed on both CSF and postmortem blood samples. All these samples were analyzed by Laboklin, Bad Kissingen, Germany. Immunohistochemistry for rabies virus antigens was carried out on selected central nervous system (CNS) sections using monoclonal anti-rabied FITC-conjugated antibodies (Sifin diagnostics GmbH, Berlin, Germany).

For fox No. 2, brain tissue was assessed for the presence of herpesviruses, flaviviruses, adenoviruses, paramyxoviruses, TBEV, and bornaviruses through consensus-nested PCRs and RT-PCRs at the Institute of Virology, Leipzig University. For paramyxoviruses, additional tissue samples—including trachea, lung, bladder, kidney, intestines, and stomach—were tested. Rabies virus detection was performed using immunofluorescence with monoclonal anti-rabies FITC-conjugated antibodies (Sifin Diagnostics GmbH, Berlin, Germany). Suid herpesvirus 1 was assessed via PCR at the State Research Institute for Public Health and Veterinary Medicine in Saxony. Additionally, *Neospora caninum* and *Toxoplasma gondii* were screened via PCR in brain tissue at the Institute of Parasitology, Leipzig University. Microbiological culture of brain tissue was performed at the State Research Institute for Public Health and Veterinary Medicine Saxony.

A summary of pathogen screening, including results, is presented in Table 1.

**Table 1:** Summary of pathogen screening for fox No. 1 (silver fox, *Vulpes vulpes*) and fox No. 2 (polar fox, *Vulpes lagopus*).

Pathogen	Case 1 (Silver fox, *Vulpes vulpes*)	Case 2 (Polar fox, *Vulpes lagopus*)
*Tick-Borne Encephalitis Virus* (TBEV)	Antibody (Ab) testing in serum and CSF: negative	RT-PCR on brain tissue: negative
Morbillivirus/Paramyxoviruses	Ab in serum: negative Immunohistochemistry on CNS tissue: negative	Consensus-nested PCR on brain and multiple organs: negative
Rabies virus	Immunohistochemistry on CNS tissue: negative	Immunofluorescence on brain tissue: negative
*Suid herpesvirus 1*	Not performed	PCR on brain tissue: negative
*Canine adenovirus 1/ adenoviruses*	Ab in CSF: negative	PCR on brain tissue: negative
Bornaviruses	Not performed	PCR on brain tissue: negative
*Influenza A virus*	Immunohistochemistry on CNS tissue: negative	Not performed
*Encephalitozoon cuniculi*	Ab in serum: negative	Not performed
*Borrelia* spp.	Ab in serum: negative	Not performed
*Neospora caninum*	Ab (serum and CSF) and PCR on CSF: negative	PCR on brain tissue: negative
*Leptospira* spp.	PCR on EDTA blood: negative	Not performed
*Anaplasma phagocytophilum*	PCR on CSF: negative	Not performed
Bacterial culture	CSF and heart blood culture: *Cutibacterium* spp.	Brain culture: low to moderate growth of *Pseudomonas* spp., *E. coli*, coagulase-negative *Staphylococcus* spp., *Enterococcus* spp.
Fungal staining	PAS and Ziehl-Neelsen stains on CNS and lung tissue: no fungal or acid-fast organisms detected	Not performed
Next-Generation Sequencing (NGS)	Brain tissue metagenomic RNA sequencing: no mammalian viral pathogens detected	Brain tissue metagenomic RNA sequencing: no mammalian viral pathogens detected

Ab, Antibody testing; PCR, Polymerase chain reaction; RT-PCR, Real-time polymerase chain reaction; CNS, Central nervous system.

### 2.6 Next-generation sequencing

Frozen brain tissues (approximately 60 mg, location not further classified) obtained at necropsy were homogenized in 500 μL phosphate- buffered saline (PBS) solution using ceramic beads in a FastPrep-24 5G homogenizer (MP Biomedical), and then centrifuged at 12,000 RCF for 5 min. RNA was extracted using QIAamp Viral RNA Mini Kit (Qiagen, Hilden, Germany) as per manufacturer’s instructions and quantified using an RNA HS assay (Thermo Fisher Scientific, Waltham, MA, United States). RNA integrity was assessed by estimation of the RIN value with an Agilent RNA 6000 Pico Kit (Agilent Technologies, Santa Clara, CA, United States) in an Agilent Bioanalyzer 2200 (Agilent Technologies, Santa Clara, CA, United States). Metagenomic sequencing was performed by Novogene using a high-throughput sequencing NovaSeq™ X Plus platform (Illumina, San Diego, CA, USA) with 12 GB raw output generated per sample. Bioinformatics analysis of the raw FASTQ sequencing data was carried out using the CZ ID open-source pipeline (6), resulting in the recovery of 18,028,222 reads and 955,744 reads from fox No. 1 and 2, respectively, which passed host and quality filters.

## 3 Clinical report

### 3.1 History

Upon arrival in the rescue facility, foxes underwent a four-week quarantine, clinical examinations, which were unremarkable, received routine vaccinations against canine distemper virus, parvovirus, canine adenovirus, rabies, leptospirosis, and parainfluenza (Nobivac® SHPPi, Merck & Co., Inc., Rahway, NJ, United States) which were boostered after 4 weeks using VERSICAN® Plus Pi/L4 (Zoetis Schweiz GmbH, Delémont). Deworming was performed using Milbemycin-Oxim and Praziquantel (Milpro Hund M 12.5 mg/125 mg, Virbac AG, Glattbrugg, Switzerland). Their diet included commercial dog food, fresh meat with different meat sources depending on availability (source and type unknown), and vegetables.

Of 13 foxes (3 males, 2 females, 8 of unknown sex), 12 exhibited neurological signs and progressive deterioration over several months. Nine foxes were adults, and four were cubs a few months old, all originating from a single litter. The foxes developed signs of systemic illness (obtundation, *n* = 12/13; elevated body temperature, *n* = 12/13; diarrhea, *n* = 1/13; dyspnoea, *n* = 5/13) nine to 12 months after arrival at the rescue center. Two days to 6 months later, clinical signs of encephalopathy appeared, including obtundation (*n* = 12/13), circling (*n* = 12/13), and ataxia (*n* = 12/13). Radiographs and blood work of 12 animals were reported unremarkable or suggested unrelated diseases (elevated kidney values, likely due to urolithiasis), except for mild leucocytosis (*n* = 2/13, unknown in *n* = 11/13). The foxes either died or were euthanized after days to weeks due to severe clinical signs.

Eleven foxes were treated with prednisolone (1 mg/kg, oral), amoxicillin-clavulanic acid (20 mg/kg, oral), and metamizole (20 mg/kg, oral), but none improved. Only one survived, later dying under anesthesia for urolithiasis surgery 2 years later. This fox showed marked vitamin profile changes: elevated B2, B9, B12, and D, and low B6, for which it received supplementation. None of the other foxes displayed significant changes in vitamin levels and were therefore not treated with supplements.

### 3.2 Case 1: silver fox (*Vulpes vulpes*)

#### 3.2.1 History

The two-year-old intact female silver fox (*Vulpes vulpes*), weighing 5.20 kg, was referred to the Department of Small Mammal, Reptile and Avian Medicine and Surgery of the University of Veterinary Medicine Hannover, Germany a few days after showing symptoms.

#### 3.2.2 Clinical evaluation

At clinical examination, no significant abnormalities were detected. The neurological examination revealed obtundation, disorientation, lower back carriage, mild ambulatory paraparesis, and mild reduced proprioception in the hindlimbs more pronounced on the left. Menace responses were bilaterally absent, with a normal pupillary light reflex and bilaterally absent lateral palpebral reflexes, while median palpebral reflexes were normal. Assessment of postural reactions was limited due to the fox’s aggressiveness. Spinal reflexes were unremarkable. The fox exhibited proximal thoracic and lumbar hyperesthesia during palpation. Neuroanatomical findings were suggestive of a multifocal, asymmetric encephalomyelopathy.

#### 3.2.3 Laboratory findings

Blood examination showed no abnormal findings. Urine analysis showed hypersthenuria (urinary specific gravity >1.050; ref. 1.008-1.012). CSF analysis showed unremarkable protein levels, total cell counts and cytology. Microbiological culture of the CSF showed no growth of aerobic or anaerobic bacteria or fungi. Blood culture revealed no growth of aerobic or anaerobic bacteria, except for a strain of *Cutibacterium acnes*, which was considered a contaminant in the sample.

#### 3.2.4 Imaging

**Figure 1:**
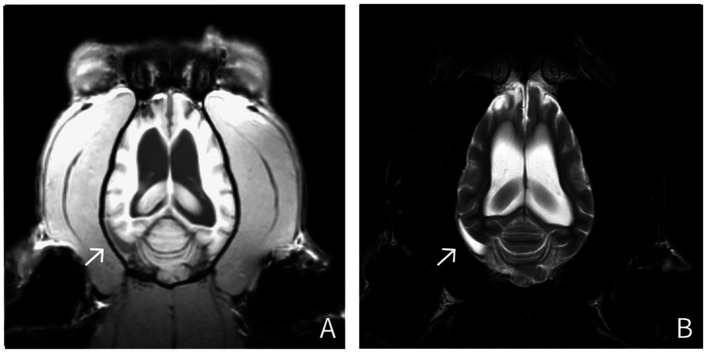
MRI findings in a silver fox (*Vulpes vulpes*) (Fox No. 1). Dorsal T1-weighted and T2-weighted images show hyperintensity on both T1- and T2-weighted sequences, along with a layered splitting of the dura mater (arrow). (A: T1-weighted, post-contrast images; B: T2-weighted images).

Radiography of the thorax and abdomen as well as abdominal ultrasound and a whole-body CT showed no abnormal findings. MRI of the brain revealed a severe dilation of the ventricular system, with incomplete suppression of the CSF on FLAIR sequences. The olfactory recesses were bilaterally severely dilated, causing a significant mass effect, characterized by compression of adjacent brain parenchyma and distortion of the normal intracranial architecture. Severe thickening of the dura mater was observed, characterized by T2w hyperintensity and splitting of the dura mater in a layered pattern, particularly on the right side and around the right temporal lobe. The dura mater also demonstrated marked contrast enhancement (Figure 1). The inter-thalamic adhesion appeared triangular and ventrally flattened. Ill-defined, irregularly marginated lesions were observed in the right and left frontal lobes and the right piriform lobe. These lesions appeared hyperintense on T2w and FLAIR images, and isointense on post-gadolinium T1w images, involving both gray and white matter. No intra-axial contrast enhancement was present. The radiological diagnosis was severe meningoencephalopathy and moderate generalized ventriculomegaly.

Under anesthesia, the fox suffered from cardiorespiratory arrest and could not be resuscitated.

#### 3.2.5 Pathological examination and findings

**Figure 2:**
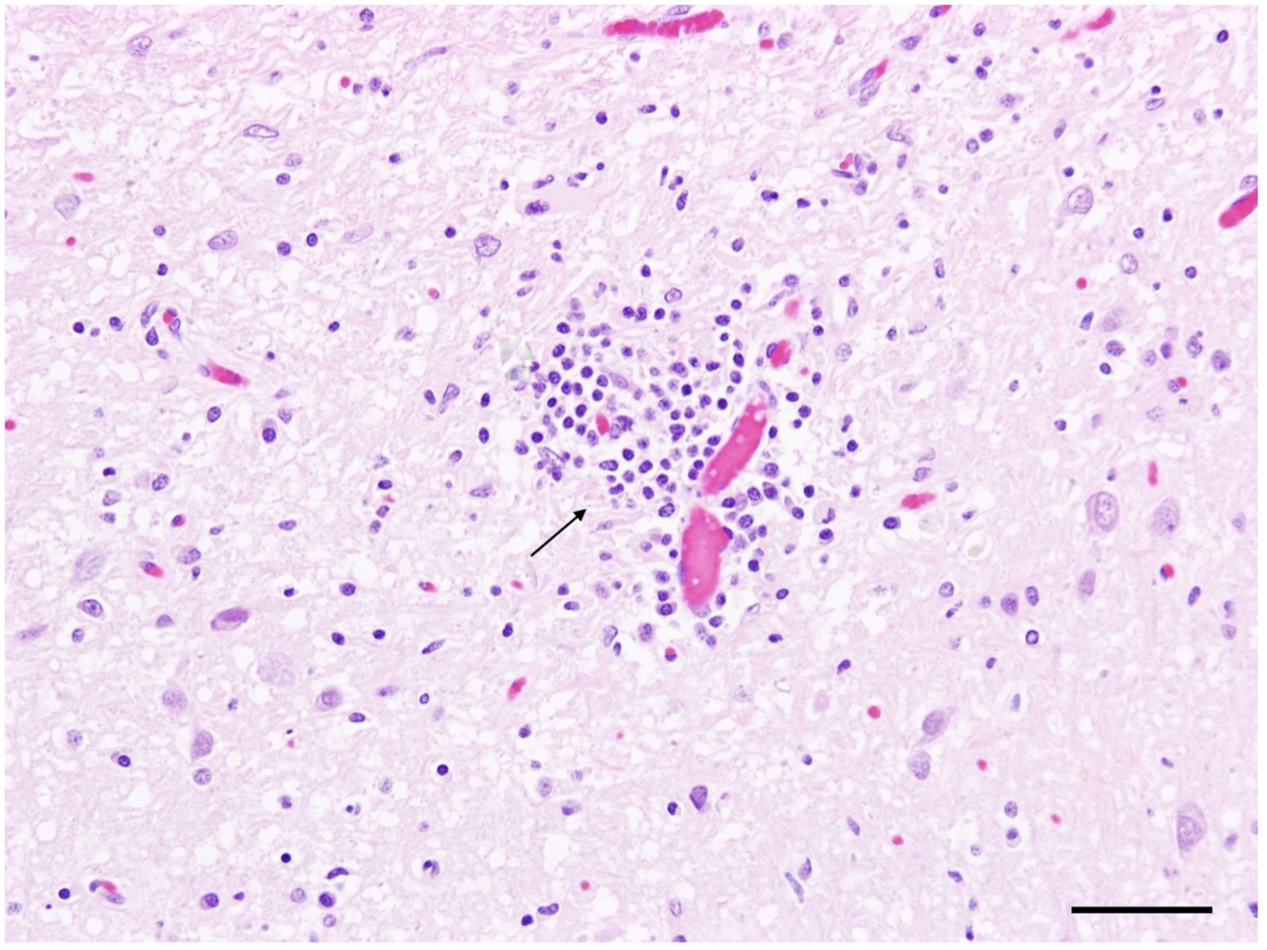
Silver fox (*Vulpes vulpes*) (Fox No. 1), brain. Mild lymphohistiocytic perivascular encephalitis in the cerebrum (arrow). Hematoxylin and eosin (H&E) stain, scale bar = 50 μm.

At gross examination, the fox was in moderate body condition with no significant lesions other than agonal changes. Histologically, there was mild, multifocal lymphohistiocytic meningoencephalitis involving the cerebrum, cerebellum, and brainstem, with a focal, severe encephalitic lesion in the cerebrum (Figure 2). The spinal cord exhibited mild, multifocal non-suppurative meningitis and myelitis, along with moderate degenerative changes in the cervical and thoracic funiculi. Mild to moderate eosinophilic inflammation was observed in the lungs, nasal cavity, urinary bladder, intestine, mesenteric lymph nodes, and skeletal muscle. Lymphoid tissues displayed follicular hyperplasia, plasmacytosis, and sinus histiocytosis. Sarcosporidian cysts were identified in skeletal muscle without associated inflammation. Immunohistochemistry for influenza A virus, canine distemper virus, and canine adenovirus was negative. Next-generation sequencing also failed to detect any mammalian-specific viral pathogens.

Pathomorphological diagnosis included a multifocal, predominantly lymphohistiocytic inflammation of the central nervous system with associated spinal cord degeneration, systemic eosinophilic inflammation, and no evidence of identifiable infectious agents by histology, immunohistochemistry, or next-generation sequencing.

### 3.3 Case 2: polar fox (*Vulpes lagopus*)

#### 3.3.1 History

A one-year-old castrated male polar fox (*Vulpes lagopus*), weighing 5.06 kg, was submitted to the Institute of Veterinary Pathology, Faculty of Veterinary Medicine, Leipzig University, following the onset of neurological signs consistent with the clinical presentation described in section 3.1. Euthanasia was elected due to progressive neurological deterioration and poor overall clinical condition.

#### 3.3.2 Pathological examination and findings

The fox presented with poor body condition. Despite typical agonal changes, no macroscopic changes were found.

**Figure 3:**
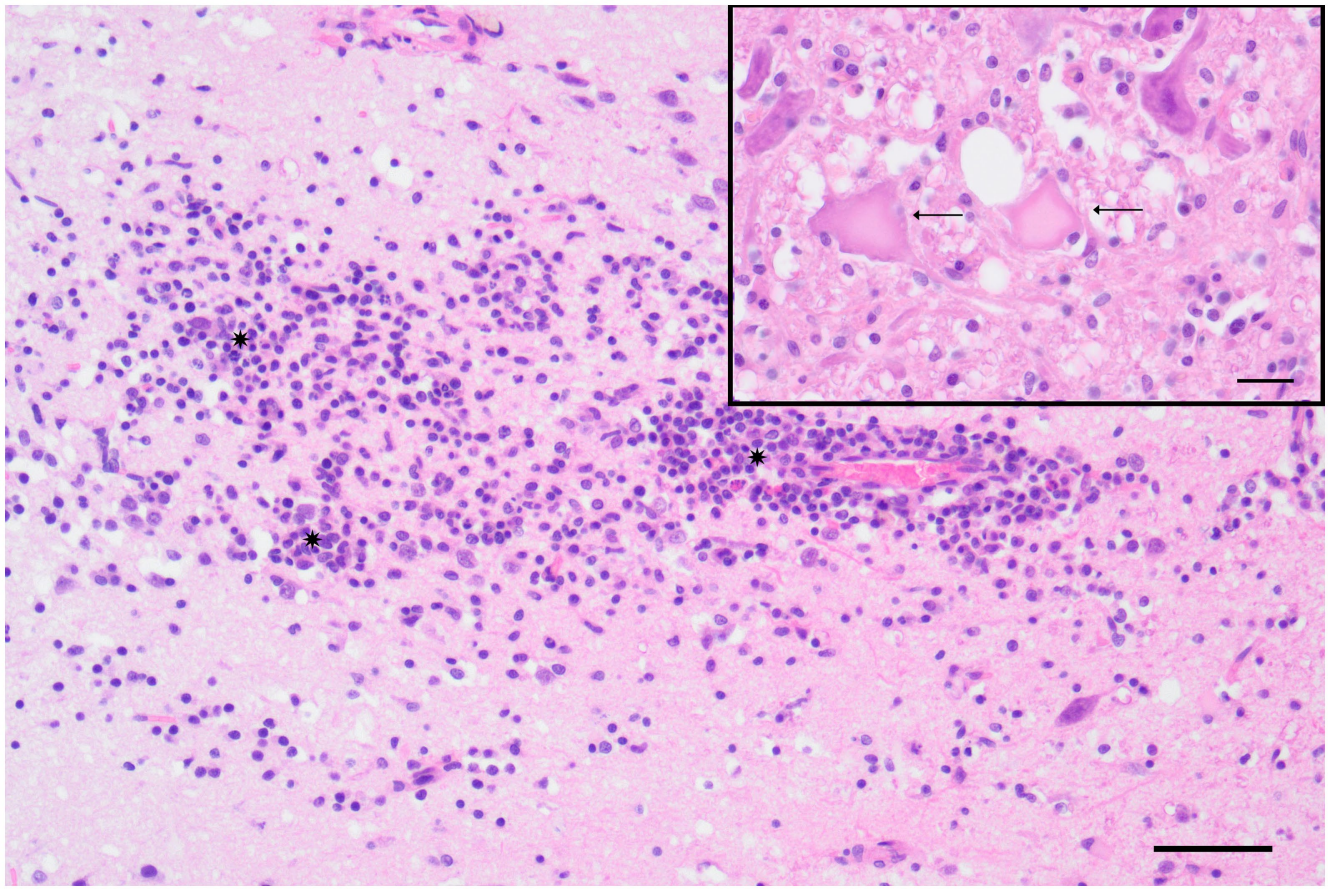
Polar fox (*Vulpes lagopus*) (Fox No. 2), brain (cerebrum), lymphohistioplasmacytic polioencephalitis characterized by the presence of a cluster of lymphocytes, histiocytes, and plasma cells (*) infiltrating the gray matter of the cortex at the level of the corpus striatum. Hematoxylin and eosin (H&E) stain, scale bar = 50 μm. Inset: Detail of neuronal necrosis, showing areas of neuronal cell death with cytoplasmic and nuclear changes (arrow) in the cortex at the level of the hippocampus. H&E stain, scale bar = 20 μm.

Microscopically, there was mild to severe, chronic, multifocal, lymphohistioplasmacytic polioencephalitis (Figure 3) with varying degrees of leukoencephalitis, meningitis, microgliosis, and neuronal necrosis (Figure 3, Inset). Spheroid formation was visible in the olfactory bulb, cerebral cortex, striatum, thalamus, hippocampus, cerebellum, and brainstem. The severity of lesions decreased progressively from rostral to caudal. The cervical spinal cord exhibited mild to moderate, multifocal, subacute to chronic, multifocal, lymphohistioplasmacytic poliomyelitis with microgliosis.

The liver exhibited mild, oligofocal, lymphohistiocytic portal infiltration.

Infectious tests were negative. Microbiological culture of the brain revealed low to moderate levels of *Pseudomonas* sp., *E. coli*, two different coagulase-negative *Staphylococcus* sp., *Enterococcus faecium* and *Enterococcus faecalis*. No mammalian specific viral pathogens were detected following NGS analysis.

A summary of the pathogen screening for both foxes is presented in Table 1.

## 4 Discussion

This case series presents clinical and necropsy findings of foxes with meningoencephalomyelitis of unknown origin. In canine and feline medicine MUO is an established term describing sporadic cases of non-infectious and most likely auto-immune mediated inflammatory diseases of the CNS (7). In contrast, reports of meningoencephalitis in wild carnivores have predominantly focused on infectious etiologies, particularly those of major relevance to wildlife health such as rabies virus, canine distemper virus, and emerging threats like highly pathogenic avian influenza (H5N1) (1, 9). It has long been suspected that MUO might be caused by a thus far undetected infectious agent (5). However, after more than five decades of research, it is becoming increasingly evident that MUO in dogs is unlikely to be caused by an infectious agent. This conclusion is supported by the consistent failure to identify any such pathogens using advanced techniques like next-generation sequencing (NGS) (7, 8, 10).

NGS enables the detection of viral DNA or RNA without prior knowledge of the specific virus being targeted (11). NGS has been used to detect Batai virus encephalitis in the brain tissue of harbor seals (12) or rustrela virus in cats with lymphohistiocytic meningoencephalomyelitis (13). Despite this promising approach, NGS did not reliably detect any viral RNA that could be associated with the disease observed in the silver and polar fox under investigation.

Although bacteria can cause meningoencephalomyelitis in various species, it is uncommon for them to produce a purely lymphohistiocytic meningoencephalomyelitis with normal CSF parameters—even under pretreatment with amoxicillin-clavulanic acid and prednisolone—especially when severe clinical signs are visible (10–12). The gold standard for diagnosing bacterial meningoencephalitis in humans typically involves bacterial culture or PCR testing of CSF (13, 14). In both foxes, bacterial cultures revealed a diverse array of bacterial species, most of which likely reflect environmental contamination during sample collection (15, 16). In case No. 2, the polar fox, *Psychrobacter* spp. and *Acinetobacter* spp. were isolated, environmental bacteria commonly found in marine settings and mammalian microbiota. Given their rare association with CNS infections (15, 17, 18), especially in healthy hosts, their presence likely reflects environmental contamination rather than a causative role in the fox’s neurological disease.

The possibility of a “hit-and-run” infection, where the pathogen is no longer present in the tissue but leaves lasting effects, could explain the negative examination for significant infectious agents (19). In humans, post-infectious autoimmune encephalitis is well-documented following infections with viruses like measles, mumps, or rubella, where low viral burden makes pathogen detection challenging (20, 21). Metagenomics still faces challenges, including limited sensitivity, high costs, and issues with bioinformatic standardization and data interpretation (21). However, host DNA depletion and hybridization-based methods can improve detection and help rule out infectious causes, supporting investigation into autoimmune mechanisms in wild carnivores (22). Encephalitis might occur secondary to toxic or metabolic disease, but the asymmetrical and painful clinical signs make intoxication and metabolic disorders unlikely (23–25).

Non-suppurative meningoencephalomyelitis without an identified infectious agent is well known in dogs, where the condition is named MUO (26). MUO is thought to result from a combination of genetic predisposition and environmental triggers (5, 7) and encompasses several histopathological subtypes (7). The histopathological and imaging findings in the foxes described here do not match any specific MUO subtype (7). But even in dogs, MUO cases present very heterogeneous, especially regarding the suspicion that more subtypes of MUO exist, which are not yet fully understood such as canine lymphohistiocytic meningoencephalitis with vasculitis (27) or auto-antibody encephalitis as seen in the death of the polar bear ‘Knut’ at the Berlin Zoo in 2011 (28).

As in dogs, the pathogenesis of MUO in wild carnivores may also be multifactorial (5). In human and, more recently, veterinary medicine, it seems that the development of the immune system is fundamentally influenced by environmental factors in early childhood (29). Human individuals who grow up closer to the equator—where sunlight exposure is greater—tend to have lower rates of multiple sclerosis (30). Fur-farmed foxes, akin to mostly indoor dogs, have limited outdoor access and thus reduced sunlight exposure. This diminished contact to sunlight during critical developmental stages may predispose them to autoimmune disorders.

Based on the “hygiene hypothesis,” early-life exposure to diverse pathogens may promote immune self-regulation in humans and pets (31). Captive foxes likely have reduced exposure to environmental antigens compared to their wild counterparts. As a result, their immune systems may lack the natural modulation provided by frequent microbial encounters (32), potentially leading to dysregulation and increased risk of autoimmunity.

Despite being from different species, both foxes originated from fur farms with a narrow genetic background, which could increase the likelihood of a genetic predisposition—similar to that reported in Pugs, Maltese, and Chihuahuas (39).

The foxes described here mostly show first clinical signs within 1 year after being rehomed from the fur farms to the rescue center. It might be, that transport stress and vaccination challenged their immune system making them prone to normal infections. Many autoimmune diseases are triggered by a previous rather harmless infection, activating the immune system and due to missing regulatory mechanisms might subsequently lead to autoimmune disease in predisposed animals (33). This multifactorial pathogenesis could also explain the limited response to corticosteroid treatment—commonly effective in MUO cases in dogs and cats—as immunosuppression might have exacerbated an undetected underlying infection, allowing it to progress.

Taken together, restricted microbial and sunlight exposure in early puppyhood coupled with limited genetic diversity and environmental stressors may have created an immunological environment leading to the emergence of autoimmune disease once a trigger was introduced. To better understand these complex immunological processes in wildlife, future studies should incorporate comprehensive assessments, including cytokine profiling and other immune function analyses. The cases of meningoencephalomyelitis in the two foxes presented in this report highlight the significant challenges faced in diagnosing and understanding CNS diseases in wild carnivores.

## Data Availability

The raw data supporting the conclusions of this article will be made available by the authors, without undue reservation. Requests to access the datasets should be directed to Natalie Steiner (Natalie.steiner@tiho-hannover.de).
